# Asymmetry of the anterior ethmoidal artery in relation to the anterior skull base: a population-based study of 500 arteries^☆^

**DOI:** 10.1016/j.bjorl.2024.101412

**Published:** 2024-03-01

**Authors:** Bernard Beraldin, Gustavo Rassier Isolan, Lucas Rodrigues Mostardeiro, Vagner Antonio Rodrigues Silva, Joel Lavinsky

**Affiliations:** a Santa Casa de Misericórdia de Porto Alegre, Porto Alegre, RS, Brazil; b Faculdade Evangélica Mackenzie do Paraná, CEANNE Brasil, Porto Alegre, RS, Brazil; c Universidade de Campinas (Unicamp), Faculdade de Ciências Médicas (FCM), Departamento de Otorrinolaringologia, Cirurgia de Cabeça e Pescoço, Campinas, SP, Brazil; d Universidade Federal do Rio Grande do Sul (UFRGS), Programa de Pós-graduação em Cirurgia, Porto Alegre, RS, Brazil

**Keywords:** Epistaxis, Skull base, Computed tomography, Paranasal sinuses

## Abstract

**Objective:**

To analyze variability in the distance between the Anterior Ethmoidal Artery (AEA) and the anterior Skull Base (SB), as well as the frequency of lateral asymmetry in a Latin American population using computed tomography.

**Methods:**

A total of 250 computed tomography scans of paranasal sinuses in coronal reconstruction (500 AEAs) were analyzed. After determining the image with the best anatomical view of the artery, the distance between its midpoint and the ethmoidal roof was measured, and the images were independently interpreted by 2 physicians.

**Results:**

Of the 500 AEAs, 279 (55.8%) adhered to or passed through the SB at a distance of 0 mm. A total of 221 AEAs (44.2%) were at some distance from the SB, of which 107 (48.4%) were on the right side, ranging from 1.18 to 6.75 mm, and 114 (51.5%) were on the left side, ranging from 1.15 to 6.04 mm. The overall mean distance between the AEA and SB was 1.22 (SD = 1.57) mm, increasing to 2.77 (SD = 1.14) when the arteries adhered to the SB were excluded. Seventy-six individuals (30.4%) had a lateral distance variation > 1 mm.

**Conclusion:**

Our study includes the largest sample of AEA analyzed with computed tomography scans of paranasal sinuses. There was some distance between the AEA and SB in almost half the patients, and we found a high rate of lateral variability >1 mm.

**Level of evidence:**

Level 3.

## Highlights

• We analyzed the largest sample of AEA with computed tomography scans.
• First study of the distance between the AEA and SB using computed tomography.
• We analyzed AEA lateral asymmetry within the same individual.

## Introduction

Endoscopic endonasal surgery requires surgeons to work in narrow spaces with extensive vascularization while limited by structures such as the orbit and the Skull Base (SB).^1^ The success of frontal sinus surgery depends on complete removal of bony septations in the recess of this sinus, although surgeons must also be aware of complications, such as cerebrospinal fluid leak and Anterior Ethmoidal Arterial (AEA) bleeding.^2^

The AEA is a branch of the ophthalmic artery, which is a branch of the internal carotid artery. It crosses 3 cavities along its path: the orbit, the ethmoidal labyrinth, and the anterior cranial fossa. In its intranasal course, the artery is usually found inside a single bone canal, called the anterior ethmoidal canal, which may or may not present dehiscence. It passes between the superior oblique and medial rectus eye muscles before leaving the orbit via the anterior ethmoidal foramen (situated in the fronto-ethmoidal suture).^3^ The AEA is indirectly identified through reference points such as the notch in the medial wall of the orbit (i.e., the anterior ethmoidal foramen) using computed tomography, since it more clearly indicates bone structures than other imaging modes.^4^

The proximity of the ethmoidal arteries to the SB is relevant in paranasal sinus surgery. Paranasal anatomy varies significantly laterally and among different individuals.^5^ There are some studies regarding measurements of the distance between the AEA and the SB, but studies on the lateral variation of the AEA and the SB are scarce and present discrepant results, varying between 4% and 30%.^6^, ^7^ Recognizing these variations prior endoscopic sinus surgery helps minimize the risk of damaging the artery, especially when it is below the SB,^8^ considering that inadvertent damage can cause complications, such as profuse bleeding, and cerebrospinal fluid rhinorrhea, while the artery’s retraction to the intraorbital region can cause amaurosis due to orbital hematoma.

Although several radiographic and cadaveric studies have increased our understanding of the characteristics of the AEA and the anatomical relationships within the ethmoid sinus, more information regarding the distance from the SB and actual differences between populations is needed, as well as the accuracy of AEA location. Thus, the importance of a study with a sample of 500 specimens is observed. In 2017, Poteet et al.^6^ reported a lack of studies on the relationship between SB height and distance from the AEA, comparing the results by sex and age. Thus, in this tomographic study, we analyzed variability in the distance between the AEA and the anterior SB, as well as the frequency of lateral asymmetry.

## Methods

This cross-sectional study was conducted at Santa Casa de Misericórdia de Porto Alegre, a tertiary in the city of Porto Alegre, state of Rio Grande do Sul, Brazil, and was submitted and approved by the Ethics and Research Committee on Human Beings of Irmandade Santa Casa de Misericórdia de Porto Alegre (ISCMPA) under number 43236820.9.0000.5335. The database of the hospital’s diagnostic imaging center was used.

The inclusion criteria were individuals aged ≥ 12 years who underwent a sinus scan with computed tomography between January 2018 and December 2022. The age of 12 years was used as a cut-off because at this age the volumetric relations of the facial sinuses differ little from those of adults, especially that of the ethmoid sinus. The exclusion criteria were individuals with a craniofacial anomaly, nasosinusal tumors, chronic rhinosinusitis with nasal polyps, trauma to the SB or face, and previous surgery in the paranasal sinuses or SB. Sampling was by convenience. 250 computed tomography scans of paranasal sinuses in coronal reconstruction (500 AEA) of healthy individuals were analyzed. This number of CT scans was used considering that it was based on sample size calculation and with a safety surplus to consider possible losses in the analysis of radiological images. The scans were acquired with an Optima 540 computed tomography system with 16 transducers (GE HealthCare, Boston, MA, USA), a 250 mm field of view, a resolution of 512 × 512 pixels, 0.625 mm spacing between slices, and a slice thickness of 0.625 mm. The protocol was based on a regime of 100 kV (fixed) and 200 mAs (modulated) and reconstruction using a bone filter (windowing 2000 width and 400 height) and a soft filter (windowing 260 width and 60 height). All data were anonymous and accessible only to members of the research team. The data were pooled, and participants were not individually identified.

**Figure 1:**
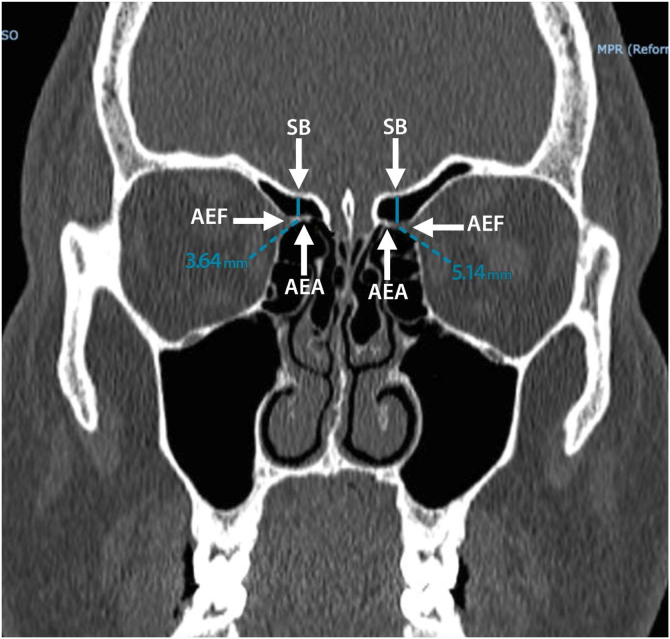
Computed tomography in coronal reconstruction showing the distance between the AEA and the SB. AEA, Anterior Ethmoidal Artery; SB, Skull Base; AEF, Anterior Ethmoidal Foramen.

**Figure 2:**
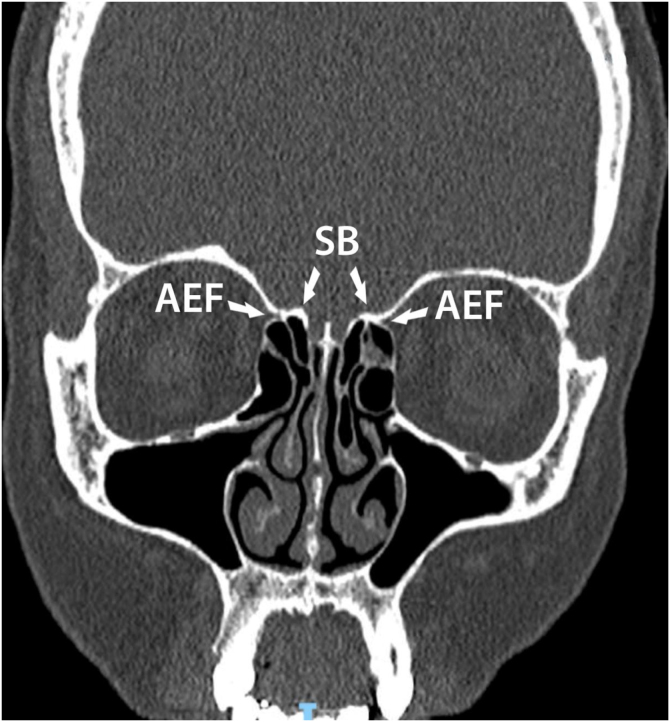
Computed tomography in coronal reconstruction showing anterior ethmoidal artery adhered to the skull base. SB, Skull Base; AEF, Anterior Ethmoidal Foramen.

The images were reconstructed in the coronal plain in the PACS system through which it was possible to perform measurements from AEA to SB. The AEA was identified using the anterior ethmoidal foramen as an adjacent bony landmark. When possible, the path of the anterior ethmoidal canal was identified, visualized as a tubular structure with an oblique course on the roof of the ethmoidal sinus. After determining the image with the best anatomical view of the artery, the distance between its midpoint and the ethmoidal roof, i.e., the anterior SB, was measured using a Carestream Vue Motion post-processing station (Carestream Health, Inc., Rochester, NY) (Figure 1, Figure 2). The arteries were analyzed independently, i.e., the same tomographic slice was not always used for both sides.

All images were independently interpreted by 2 physicians (an Otolaryngology-Head and Neck Surgery Resident in third year of residence and a rhinologist). All measurements were made in triplicate and the mean value was used for data analysis. When any difference was found between the examiners' measurements, the test was analyzed by all the researchers until consensus was reached.

The images were documented photographically, and the data were tabulated in Microsoft Excel for OSX. Statistical analyses were performed in IBM SPSS Statistics 20.0 (IBM, Armonk, NY, USA), with p-values <0.05 considered statistically significant.

## Results

This study involved a total of 250 individuals, 97 men (38.8%) and 153 women (61.2%). The mean age was 46.50 (SD = 19.3; range: 12–99) years. A total of 500 AEA were analyzed. The anterior ethmoidal foramen, identified as a notch in the medial wall of the orbit, was visualized in 100% of the cases.

Of the 500 arteries, 279 (55.8%) AEAs adhered to or passed through the SB. Of these, 143 (51.2%) were on the right side and 136 (48.7%) on the left side. This happened both bilaterally (same patient) and unilaterally (the contralateral artery was at some distance from the base of the skull). Thus, a total of 221 (44.2%) arteries were at some distance from the SB, of which 107 (48.4%) were on the right side, ranging from 1.18 to 6.75 mm, and 114 (51.5%) were on the left side, ranging from 1.15 to 6.04 mm.

When we analyzed only the arteries at some distance from the SB, the mean rose to 2.77 (SD = 1.14) mm, being 2.70 (SD = 1.16) mm on the right side and 2.83 (SD = 1.11) mm on the left. In 87 of the participants, the arteries were at some distance from the SB bilaterally, making them the best group for comparison. However, there was no significant difference between the right side (2.83 [SD1.22] mm) and the left (2.94 [SD = 1.16] mm) (Student's *t*-test for paired samples, *p* = 0.374). We also analyzed distance variation according to sex (Table 1).

**Table 1:** Distance variation among women and men.

		Overall distance in women	Overall non-zero distance in women	Overall distance in men	Overall non-zero distance in men
N	Valid	306	130	91	91
	Missing	0	176	103	103
Mean		1.16 mm	2.74 mm	1.32 mm	2.82 mm
SD		1.55 mm	1.17 mm	1.60 mm	1.11 mm
Minimum		0.00 mm	1.15 mm	0.00 mm	1.18 mm
Maximum		6.75 mm	6.75 mm	6.04 mm	6.04 mm

Analyzing lateral differences in men, the mean distance was 1.24 (SD = 1.50) mm on the right side (median: 0 [IQR 0–2.24]) and 1.39 (SD = 1.69) mm on the left (median: 0 [IQR 0–2.75]). In women, it was 1.09 (SD = 1.56) mm (median: 0 [IQR 0–2.12] on the right side and 1.22 (SD = 1.54) mm on the left (median: 0 [IQR 0–2.36]) There were no significant within-sex differences between sides (Wilcoxon test: *p* = 0.287 for men and *p* = 0.225 for women).

**Figure 3:**
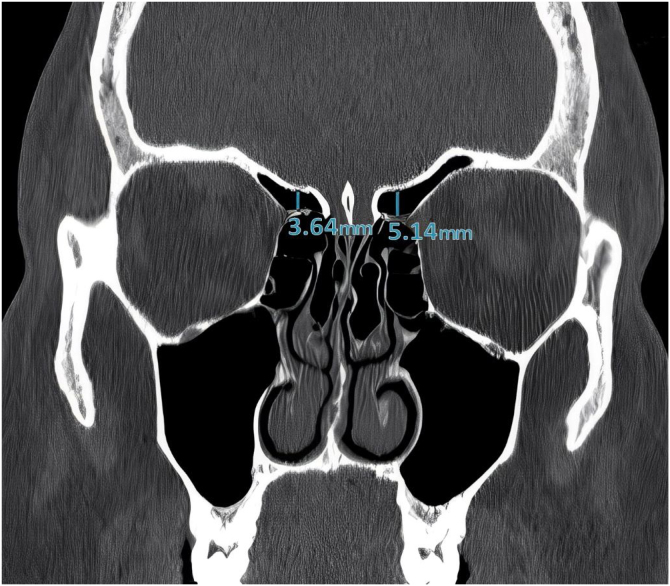
Computed tomography showing lateral distance variation.

We also calculated the percentage of individuals with a lateral distance variation > 1 mm (Fig. 3), finding a total of 76 (30.4%) individuals (48 [63.2%] women and 28 [36.8%] men) but no association with sex (Chi-Square test with Yates correction: *p* = 0.780).

Finally, we found a total of 221 (44.2%) anterior ethmoidal canals, of which 160 (32%) were complete and 61 (12.2%) were incomplete. When correlating age and distance, no significant difference was found on the right side (Pearson's correlation coefficient: *r* = 0.08, *p* = 0.418) or the left (*r* = 0.05, *p* = 0.575).

## Discussion

Although functional endoscopic sinus surgery is an effective means of treating patients with recurrent and refractory sinusitis, the procedure is not without risk of serious complications. Preoperative computed tomography offers surgeons the opportunity to prospectively identify anatomical variants that predispose patients to major surgical complications.^9^

There is considerable variability in the AEA as it leaves the orbit, crossing the ethmoidal sinus and reaching the orbit in anteromedially to reach the lateral lamella of the cribriform plate. Failure to recognize arteries that pass below the SB can result in iatrogenic injury during endoscopic endonasal surgery, which can result in important complications, such as orbital hematoma, vision loss, and secondarily cerebrospinal fluid leakage.^10^ Thus, detailed knowledge of AEA anatomy and its variations is essential to avoid complications during surgery.

In our sample, 44.2% of the AEA were at some distance from the SB. This result is similar to that of Abdullah et al.^8^ and Poteet et al.,^6^ but differs from Kho et al.^2^ and Joshi et al.,^7^ who found a higher percentage of AEA below the SB. These results might be related to ethnical differences or to the number of scans analyzed.

Comparable to the international literature, we identified 100% of the AEA by visualizing the anterior ethmoidal foramen.^4^ Thus, we consider this bone notch on the medial wall of the orbit to be a constant landmark that is preserved even in extensive pathologies of the paranasal sinuses.

In recent years, several studies have measured the distance between the AEA and the SB^5^, ^8^, ^11^ (Table 2), although none have been conducted in Latin America. Few studies have analyzed intersex differences in distance and have none assessed whether they change with age. As far as we know, our study includes the largest sample of AEA analyzed with computed tomography scans.

**Table 2:** Mean anterior ethmoidal artery distance from the skull base in other studies.

Author	Year	CTs	Results
Jang et al.	2014	78	1.32 ± 1.51 mm
Abdullah et al.	2019	126	1.93 ± 2.03 mm
El-Anwar et al.	2020	150	1.37 ± 1.98 mm
Beraldin et al.	2023	221	2.77 ± 1.14 mm

CTs, number of Computed Tomography scans.

The overall mean distance between the AEA and the SB in our sample was 1.22 (SD = 1.57) mm (range 0–6.75 mm), which, although smaller, was similar to the results of the aforementioned studies. When we analyzed only AEAs at some distance from the SB, the overall mean rose to 2.77 (SD = 1.14) mm, being 2.70 (SD = 1.16) mm on the right side and 2.83 (SD = 1.11) mm on the left, which is very similar to the results of Cascio et al.^12^ in an Italian population but unlike those of Poteet et al.^6^ in a U.S. population (Table 3). Again, these differences may be related to morphological variation between populations.

**Table 3:** The mean distance from the anterior ethmoidal artery to the skull base according to laterality in other studies, excluding arteries adhered to the skull base.

Author	Overall mean	Right	Left
Beraldin et al.	2.77 ± 1.14 mm	2.70 ± 1.16 mm	2.83 ± 1.11 mm
Cascio et al.		2.51 ± 2.54 mm	2.64 ± 2.54 mm
Poteet et al.		3.18 mm	3.85 mm

Although the position of the AEA may vary laterally within the same patient, information about this is scarce in the literature. Lateral variation occurred in 30.4% of our sample. In a similar study with a small sample (50 CTs), Joshi et al.^7^ found similar variability (30%). In a larger sample (101 CTs), albeit far smaller than ours, Poteet et al.^6^ found lateral variation in only 4%. Endonasal surgeons should include detailed bilateral study of this artery's path in the preoperative tomographic protocol to ensure a safe surgery.

## Conclusions

So, after analyzing a large number of CT scans, we can infer that there was some distance between the AEA and SB in 44 percent of patients, and we found a high rate of lateral variability >1 mm is 30 percent.

## Funding

The authors have no financial relationships relevant to this article to disclose.

## Conflicts of interest

The authors declare no conflicts of interest.
